# Clinicopathological analysis of odontogenic tumors over 22 years period: Experience of a single center in northeastern Brazil

**DOI:** 10.4317/medoral.22618

**Published:** 2018-11-21

**Authors:** Wenya-Kayse-Duarte de Medeiros, Leorik-Pereira da Silva, Pedro-Paulo-de Andrade Santos, Leão-Pereira Pinto, Lélia-Batista de Souza

**Affiliations:** 1 DDS, Department of Dentistry, Federal University of Rio Grande do Norte - UFRN, Natal, Rio Grande do Norte, Brazil; 2 DDS, MSc, PhD student, Postgraduate Program in Oral Pathology, Dentistry Department, Federal University of Rio Grande do Norte, Natal, RN, Brazil; 3 DDS, MSc, PhD, Titular Professor, Postgraduate Program in Oral Pathology, Dentistry Department, Federal University of Rio Grande do Norte, Natal, RN, Brazil; 4 DDS, MSc, PhD, Adjunct Professor, Postgraduate Program in Oral Pathology, Dentistry Department, Federal University of Rio Grande do Norte, Natal, RN, Brazil

## Abstract

**Background:**

Odontogenic tumors (OTs) are uncommon neoplastic lesions of the maxilla and mandible, which present difficult diagnosis and therapeutics. This paper aims to determine the frequency and distribution of OTs, over a period of 22 years, at a public university in Northeastern Brazil.

**Material and Methods:**

We reviewed all cases of OTs from oral pathology laboratory of Federal University of Rio Grande do Norte (UFRN), from 1996 to 2017. The tumors were classified according to the latest (2017) World Health Organization Classification of Tumors. Data on age, gender, anatomic site, symptomatology, radiographic findings and tumor size were analyzed.

**Results:**

In the analyzed period, 247 cases of OTs were diagnosed. Epithelial tumors were more common with 127 cases (51.8%). The most common tumors were ameloblastoma (n = 112 / 45.4%), odontoma (n = 89 / 36.1%) and odontogenic myxoma (n = 17 / 6.9%). Malignant odontogenic tumors were extremely rare in the studied population with only 2 cases (0.8%) of diagnosed carcinomas. These tumors were diagnosed in a wide age range, from 5 to 81 years, being more common in the second and third decades of life. In general, the mandible was the most affected anatomic site (n = 162/66%) and the mandible:maxilla ratio was of 2:1. Ameloblastoma was the tumor with the highest number of symptomatic cases (n = 26) and with the highest mean size (cm) with 4.5cm.

**Conclusions:**

Odontogenic tumors were rare in the sample studied (2.2%), with ameloblastoma and odontoma being the most common tumors. Continuous studies that show the characteristics of these lesions are fundamental, especially after modifications in the international classification.

** Key words:**Odontogenic tumors, jaw neoplasms, epidemiology, oral pathology.

## Introduction

Odontogenic tumors (OT), considered common, complex lesions in the gnathic bones, are difficult to diagnose and present a therapeutic challenge. They are neoplasms derived from the epithelial and/or ectomesenchymal tissues that are responsible for the development of teeth. The majority of these lesions represent true neoplasms and some may rarely exhibit malignant behavior, while other may present as malformations similar to the tumor (hamartomas). Furthermore, studies have shown that the distribution and frequency of these entities present geographic variations (1-3).

Bearing in mind the diversity of tumors and cysts that may arise from the odontogenic tissues, different classification systems have been published in an endeavor to define diagnostic criteria, with the purpose of uniformization of the diagnosis and treatment of patients affected. The first classification of OT was published in 1971 by the World Health Organization (WHO), and a second updated edition of the classification was published in 1992. Due to the scientific advances over the last few decades, a revision of the edition of the WHO classification of 1992 was published by Philipsen and Reichart in 2005 (2-5), and recently, the WHO established a new classification for odontogenic cysts and tumors (6,7).

According to the present classification, epithelial OT originate in the odontogenic epithelium without participation of the ectomesenchyme. This group comprises various tumors, with the ameloblastoma being one of the most important, due to their higher incidence and aggressive clinical behavior. Whereas, when the odontogenic epithelium and ectomesenchyme are involved they are classified as mixed odontogenic tumors. These in turn, may or may not present the formation of mineralized dental tissue. Odontomas are more common entities of this group, and are considered non-neoplastic developmental changes, instead of true neoplasms. In addition to these, ectomesenchymal OT occur; these are composed of elements of the ectomesenchyme, and the odontogenic myxoma is one of the most common tumors in this group.

The following were some of the main changes in the latest WHO classification of these tumors: inclusion of the primordial odontogenic tumor within the mixed tumors; inclusion of the cemento-ossifying fibroma in the group of mesenchymal tumors; inclusion of fibro-odontomas as being one of the variants of the odontoma; exclusion of the “keratocystic odontogenic tumor” and the “calcifying cystic odontogenic tumor” that return to the classification of odontogenic cysts (6,7). Various epidemiological studies conducted in different parts of the world have shown differences with regard to the frequency of OT according to the classification used (2,8-12).

Within this context, the importance is shown of continually conducting studies with an approach to the clinical characteristics and profile of incidence of OT, which are imperative in the scientific and clinical field. Therefore, the aim of this study was to determine the profile of incidence and present the main clinicopathological characteristics of this varied group of lesions, in the period covering 22 years (1996-2017) in a public oral diagnostic service in the Northeast of Brazil.

## Material and Methods

The data and histological material was recovered from the oral pathology laboratory, Federal University of Rio Grande do Norte (UFRN), Northeastern Brazil. Cases diagnosed of patients with OTs over a 22-year period (1996-2017) were used. This research was evaluated and approved by the Research Ethics Committee of UFRN according to CAAE n ° 54296816.1.0000.5537 (n ° 416/12).

To evaluate the incidence of OTs in the region the sample size was the population of Natal city in Rio Grande do Norte-Brazil according to the annual averaged populations for 2017, reported by the Brazilian Institute of Geography and Statistics as 885.180 people. Cases diagnosed histopathologically as OT were retrieved from the laboratory for review and were evaluated according to the 2017 WHO Classification of OTs (6,7). Data were analyzed for age, gender, tumor site, symptomatology (pain/swelling), tumor size, radiographic findings and histopathologic type.

After the sample was obtained, a database was generated using commercially available software (SPSS 20.0). Continuous variables were categorized to facilitate data analysis and presentation. Clinicopathological data analysis were done using the binomial test. X2 and fisher test was applied to check the statistical significance of the findings. The level of significance adopted was *p*< 0.05.

## Results

During the period studied (1996-2017), 10.970 oral and maxillofacial lesions were registered in the above-mentioned service, with 247 (2.2%) cases of OTs being diagnosed. The epithelial OT were the most common, with 127 cases (51.4%), followed by mixed OT, with 94 cases (38.1%), and mesenchymal tumors, with 24 cases (9.7%). Malignant OT were extremely rare in the studied population, with only 2 cases (0.8%) of carcinomas diagnosed.

The estimated global incidence of OT in the population of the studied city was 27.7 in every 100 thousand inhabitants. The incidence of epithelial OTs was 14.3 for every 100 thousand persons, followed by mixed odontogenic tumors (10.6/100 thousand) and mesenchymal tumors (2.5/100 thousand). Malignant tumors presented an incidence of 0.2 for every 100 thousand inhabitants.

The most common tumors in each group were as follows: ameloblastoma (n=112/45.4%), odontoma (n=89/36.1%) and odontogenic myxoma (n=17/6.9%). These tumors were diagnosed in a broad age-group, from 5 to 81 years, although the occurrence had been more common in the second and third decades of life. Malignant OTs were diagnosed in more advanced age-groups ( Table 1). Relative to gender, there was a discretely higher incidence in women (n=136/55.0%), in a male:female ratio of 1:1.2.

**Table 1:**
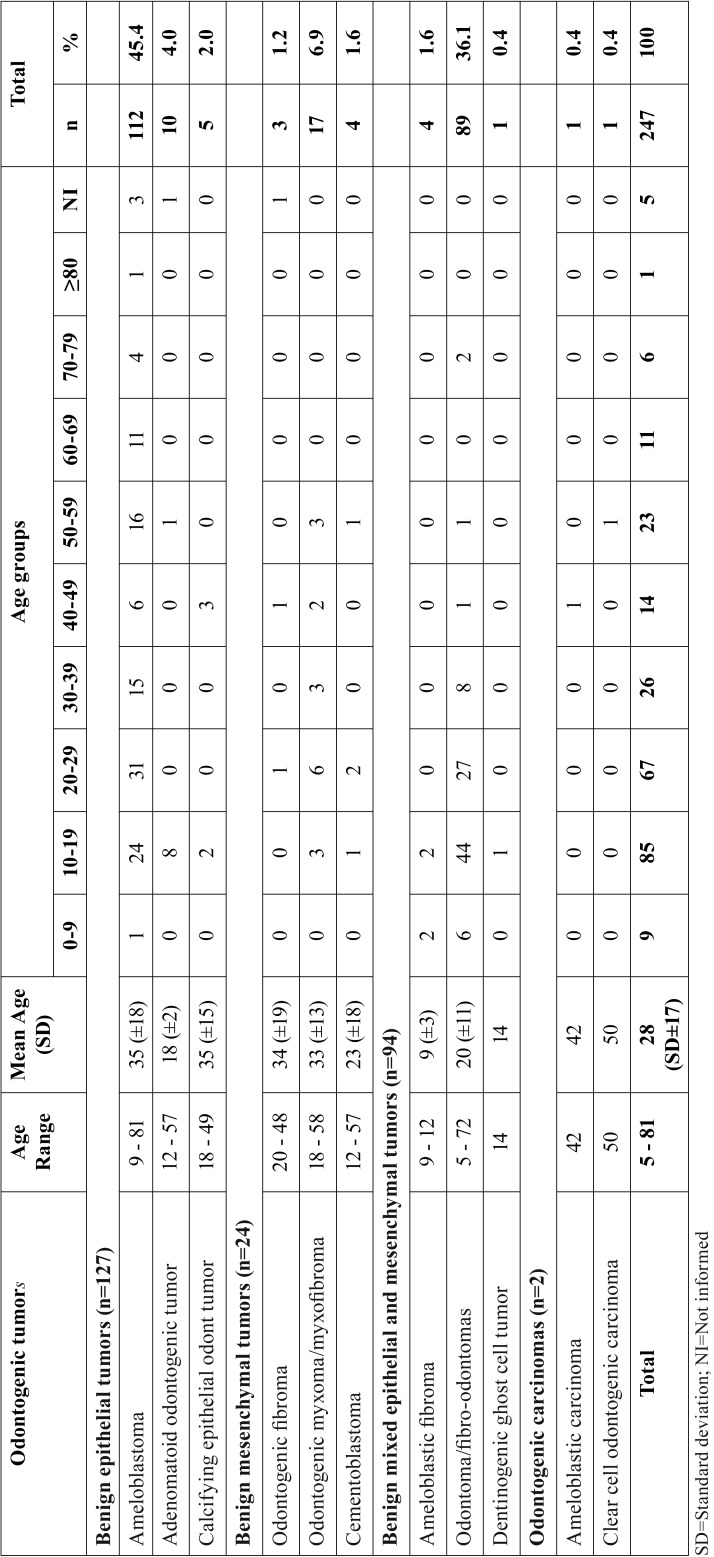
Age group distribution (decade of life) of malignant and benign odontogenic tumors.

In relation to the types of ameloblastomas found in this study (n=112), the highest prevalence was of the solid/multicystic type (n=99/88.4%), followed by the unicystic with 11 (9.8%) cases and the extraosseous/peripheral with 2 (1.8%) cases. No case of metastasizing ameloblastoma was diagnosed in the studied population.

The majority of odontogenic neoplasms were diagnosed in the mandible (n=162/66%) mainly in the posterior region (n=98/40%) in a mandible:maxilla ratio of 2:1, however, the odontoma and adenomatoid odontogenic tumor were more common in the anterior region of the maxilla ( Table 2).

**Table 2:**
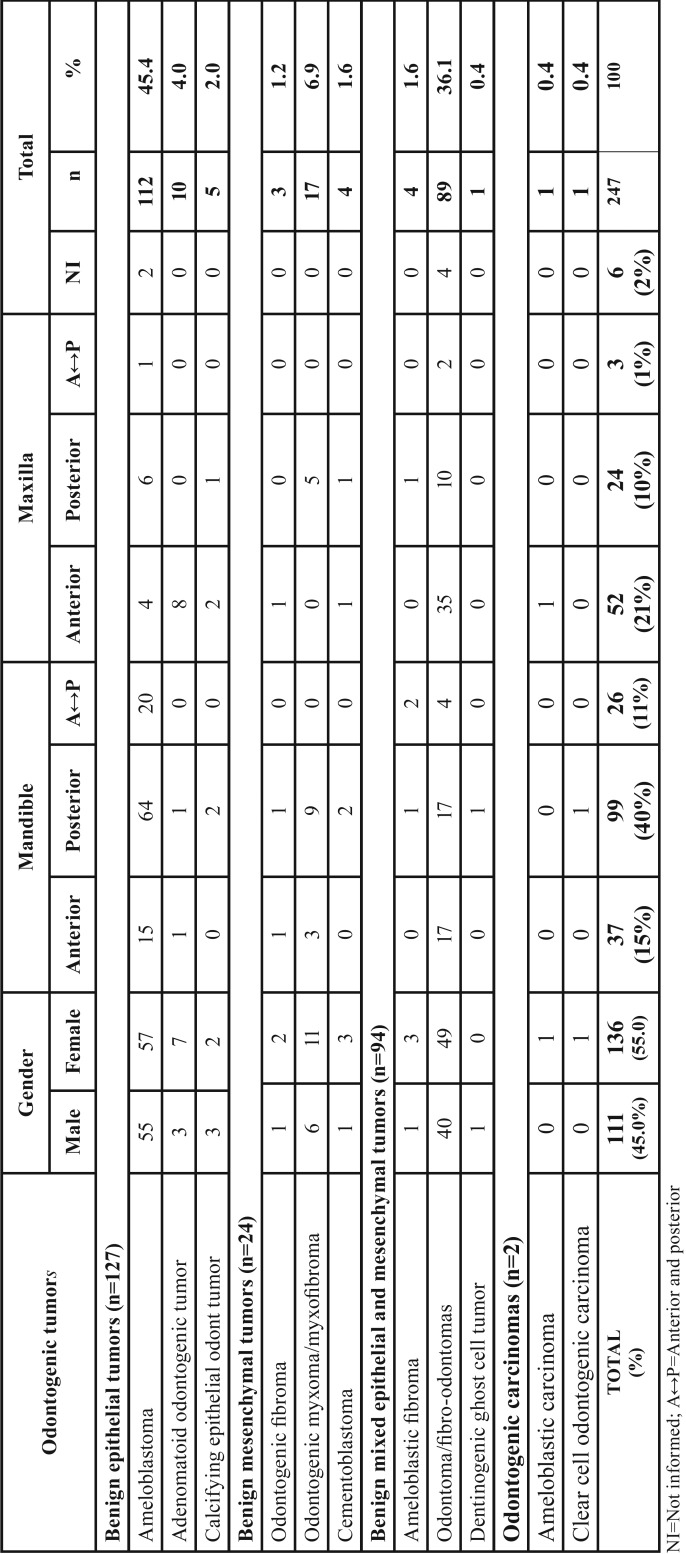
Distribution by gender and anatomic site of malignant and benign odontogenic tumors.

As regards the size of tumors, there was a variation from 0.3 to 13cm; and the ameloblastoma was the tumor that presented the largest sizes and highest mean size value, while the odontoma and cementoblastoma presented the smallest sized in centimeters. The majority of odontogenic tumors were asymptomatic (n=184/75.1%), however, the ameloblastoma was the benign tumor presenting the highest number of symptomatic cases (pain/swelling). Moreover, the two cases of malignant tumors also presented presence of symptomatology ( Table 3). No statistically significant differences were observed between OTs and the analyzed variables.

**Table 3:**
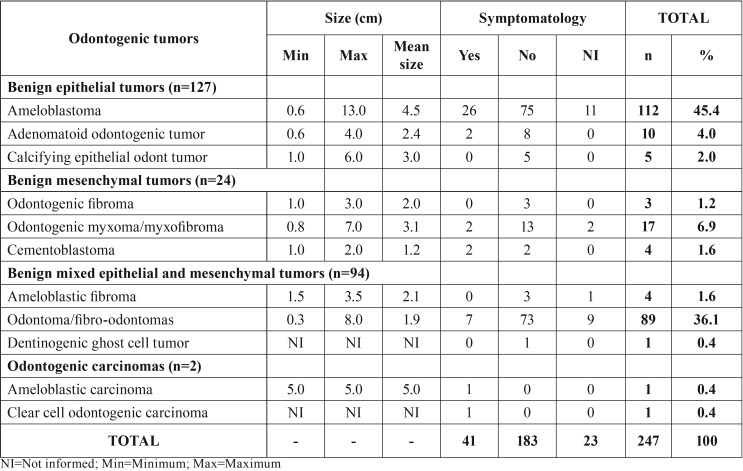
Clinical characteristics of benign and malignant odontogenic tumors.

 Table 4 shows the main radiographic findings of OTs. The lesions were mainly radiolucent and unilocular, except for odontomas and cementoblastomas that presented radiopaque radiographic appearance.

**Table 4:**
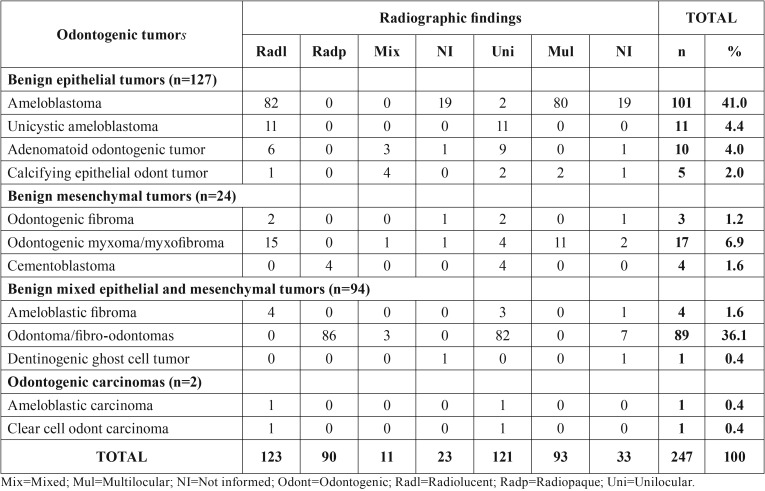
Radiographic characteristics of benign and malignant odontogenic tumors.

## Discussion

The incidence and prevalence of OTs differs according to the continent studied. In the present study, the OTs represented 2.2% of all the oral and maxillofacial lesions, corroborating the findings of researches conducted in South America, North America and Europe, which showed an incidence of between 2% to 5% of all the oral lesions diagnosed (2,9,11-14). On the other hand, studies conducted in Africa and Asia (8,10,15-17) showed a higher frequency of these lesions, affecting up to 8.9% of all the tumors diagnosed.

In addition to the geographic localization in which the study was conducted, the classification used for diagnosing the OTs may also change the profile of incidence and prevalence of these tumors. In the present study, the ameloblastoma (n=112/45.4%) followed by the odontoma (n=89/36.1%) represented the most frequent tumors. In general, according to the authors, these tumors really presented a higher incidence among all the OTs (2,8,15,18-26). Although there may be variation in occurrence among the countries, in the studies of African and Asiatic countries there was evident higher incidence of the cases of ameloblastomas (8,16-18,21,24-27).

The WHO classification in 2005 considered four types of ameloblastoma (desmoplastic, solid/multicystic, unicystic and peripheral). However, the 2017 classification modified to ameloblastoma (conventional, solid/multicystic), unicystic ameloblastoma, extraosseous/peripheral and metastasizing types. Instead of being classified as a separate entity, the desmoplastic type is now considered a histopathologic subtype of ameloblastoma, among with the follicular, plexiform, acanthomatous, granular cell, and basaloid patterns (6,7). In the present study, the solid/multicystic type was the most common ameloblastoma, whereas the extraosseous/peripheral type was rare. Several studies have been published about OT epidemiology, but few have described the types of ameloblastoma. According to some studies in India (22), Iran (18), Greece (13) and China (10) the most common ameloblastoma is the solid/multicystic type followed by the unicystic that presents a prevalence between 5 and 15%, similar to our results. On the other hand, a study conducted by Buchner *et al.* (19) reported that 46% of ameloblastomas in their series were unicystic.

In addition to these geographic differences that changed to profile of occurrence of a histological type of tumor, studies conducted after the WHO classification of 2005, in which they considered the odontogenic keratocyst a tumor; they pointed out this lesion as being the most frequent, also exceeding the frequency of the odontoma and ameloblastoma (1,2,9,10,12-14). However, in 2017, the WHO published a new classification in which they returned the odontogenic keratocyst and calcifying odontogenic cyst to the group of odontogenic cysts (6,7).

 Table 5 shows the studies conducted in Brazil after the change in the WHO classification of OTs in 2005. Of the seven studies published in the last 10 years, six of them pointed out the odontogenic keratocyst as the most common “tumor”. Irrespective of there being agreement about the classification of these lesions, over the last few years, in fact, the inclusion of these cysts as tumors in the WHO classification of 2005, not only increased the general frequency of OTs, but also led to changes in the profile of incidence and prevalence of these neoplasms all over the world (1,2,9,11,12,31,32).

**Table 5:**
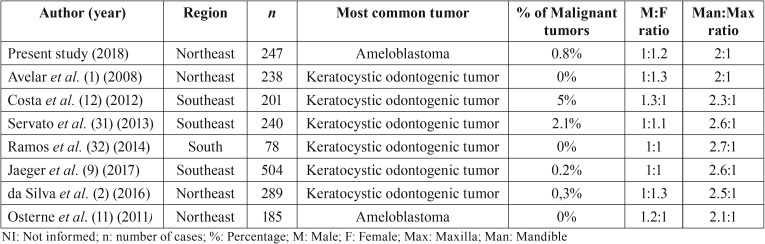
General features of the studies analyzed in Brazil (2007-2017).

Benign OTs represented the large majority of the cases in this study (n=245/99.2%). Malignant OTs were uncommon, representing only 0.85 of the total number of tumors studied (one case of ameloblastic carcinoma and one case of clear cell odontogenic carcinoma). These data are in agreement with the previous literature that pointed out a prevalence of these tumors of 0 to 5.9% of the OTs analyzed; and the ameloblastic carcinoma as the being most common malignancy (3,8-14,16,18,21,23). According to Martinez *et al.* (28) the ameloblastic carcinoma is the most common odontogenic malignancy, followed by primary intraosseous squamous cell carcinoma.

At present, the WHO classifies the cemento-ossifying fibroma as an OT; this lesion is composed of fibrous connective tissue that contains a variable mixture of bone trabeculae and concentric calcifications resembling cement (6,7). However, this origin is doubtful, because microscopically the neoplasms are identical with differentiation similar to that of cement also having been reported in the orbit, frontal, ethmoid, sphenoid and temporal bone (29). Our research group believes that there is no consensus for considering the cemento-ossifying fibroma an OT or bone neoplasm; this being so, these cases were not included in this study.

With regard to gender, a discretely higher incidence was observed in patients of the female gender, confirmed by other studies, both in Brazilian and those such as the studies of Avelar *et al.* (1) and Jaeger *et al.* (9), and those in other countries, such as the studies conducted in China, Iran and Lybia (8,18,27).

Relative to the age of patients affected by OTs, in the present study, these tumors were diagnosed in patients between 5 and 81 years of age. However, there was a peak of incidence in the second and third decades of life, data similar to those found in the literature (1-5,8-27). On the other hand, in spite of being rare the malignant OTs were diagnosed after the fifth decade of life, as is generally expected of malignant tumors in this group (28).

In this research, the anatomic localization most affected by odontogenic tumors was the mandible, with the mandible:maxilla ratio being approximately 2:1, similar to that pointed out by the literature (1-3,8-15). Nevertheless, some studies conducted in Africa (16,21,22,25) presented a higher mandible:maxilla ratio (up to 11:1) when compared with studies conducted in other continents. On the other hand, in the present study, the adenomatoid odontogenic tumor and odontoma were the most common types in the maxilla, particularly in the anterior region. These results were also found by other authors (1-3).

The majority of odontogenic tumors presented slow growth, self-limitation and were asymptomatic, and were commonly diagnosed by means of routine radiographs. Nevertheless, some tumors such as the ameloblastoma may present exacerbated growth and be symptomatic (3,21). The presence of symptomatology (pain/swelling) was infrequent in the studied sample (n=40/16%), and the ameloblastoma was the tumor that most presented symptoms. In agreement with the research of Avelar *et al.* (1) odontogenic lesions are mainly asymptomatic, however, the odontogenic keratocyst, myxoma and ameloblastoma are the lesions most associated with the presence of symptomatology.

In the present study, the size of OTs varied between 0.3 and 13 cm; and the ameloblastoma was the tumor with the largest mean size, and the cementoblastoma, the tumor with the smallest mean size. Information about the size of tumors is scarce in the literature, but our findings corroborated the findings of the study of Naz *et al.* (26), who pointed out that the size of OTs varied between 0.5 and 12.5cm at the time of diagnosis.

Interestingly, previously was performed a first study conducted by Santos *et al.* (30), at the same diagnostic center where the present study was conducted - the pathological anatomy service (Oral Pathology) of the Federal University of Rio Grande do Norte, Brazil - during the period from 1970 to 1995, in which 127 cases of OTs were found. Comparing the results of the two studies, there is evident increase in the cases of OTs diagnosed by the same Oral Pathology service. In the present study, 247 cases were found in a period of 22 years (1996-2017); that is to say double the number of tumors in a shorter time interval. We believe that the lower number of cases diagnosed in the first study was probably due to the fact that the surgical specimens were not sent for histopathological analysis. We also point out that the advance in diagnostic and therapeutic tools may be responsible for the increase in the number of cases sent for histopathological diagnosis.

## Conclusions

In addition to knowledge with respect to the biological behavior, it is extremely important for epidemiological studies to be conducted continually; studies with an approach to the general clinicopathological characteristics of tumors that occur in the maxillofacial complex, particularly when there are changes in the international classification of these tumors. Furthermore, it is necessary for health professionals to have the initiative and knowledge to perform biopsies that lead to the histopathological diagnosis, and thereby make it possible to obtain data that represent the real profile of incidence of OTs. Data such as these could contribute to alerting clinical scientists and surgeons all over the world to deal with the most varied lesions of the maxillofacial complex.
